# Bis(methyl­ammonium) tetra­sulfido­tungstate(VI)

**DOI:** 10.1107/S1600536807067682

**Published:** 2008-01-04

**Authors:** Bikshandarkoil R. Srinivasan, Christian Näther, Wolfgang Bensch

**Affiliations:** a Department of Chemistry, Goa University, Goa 403206, India; b Institut für Anorganische Chemie, Christian-Albrechts-Universität Kiel, Olshausenstrasse 40, D-24098 Kiel, Germany

## Abstract

The title compound, (CH_6_N)_2_[WS_4_], was synthesized by the reaction of ammonium tetra­sulfidotungstate(VI) with aqueous methyl­amine. The title compound is isotypic with the corresponding Mo analogue (CH_6_N)_2_[MoS_4_], and its structure consists of a slightly distorted tetra­hedral [WS_4_]^2−^ dianion and two crystallographically independent methyl­ammonium (MeNH_3_) cations, all of which are located on crystallographic mirror planes. The tetra­sulfidotungstate anions are linked to the organic cations *via* hydrogen-bonding inter­actions.

## Related literature

Previous reports give details of the structural characterization of several organic ammonium tetra­sulfidotungstates containing organic cations derived from a variety of amines (Srinivasan, Naik *et al.*, 2007 ▶ and related literature cited therein; Srinivasan, Girkar & Raghavaiah 2007 ▶ and related literature cited therein). The title compound is isotypic with (NH_4_)_2_[WS_4_], (Srinivasan *et al.*, 2004 ▶), Rb_2_[WS_4_] (Yao & Ibers, 2004 ▶), Cs_2_[WS_4_] (Srinivasan, Näther & Bensch 2007 ▶) and (CNH_6_)_2_[MoS_4_] (Srinivasan, Näther *et al.*, 2006 ▶). For related literature, see: Bondi (1964 ▶); Jeffrey (1997 ▶).
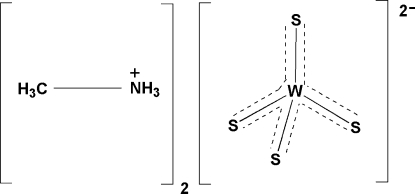


## Experimental

### 

#### Crystal data

(CH_6_N)_2_[WS_4_]
*M* *_r_* = 376.23
Orthorhombic, 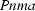

*a* = 9.5591 (10) Å
*b* = 7.0006 (7) Å
*c* = 15.734 (2) Å
*V* = 1052.9 (2) Å^3^
*Z* = 4
Mo *K*α radiation
μ = 11.70 mm^−1^
*T* = 170 (2) K
0.12 × 0.06 × 0.04 mm

#### Data collection

Stoe IPDS1 diffractometer
Absorption correction: numerical (*X-SHAPE*; Stoe & Cie, 1998 ▶) *T*_min_ = 0.441, *T*_max_ = 0.631
9418 measured reflections
1355 independent reflections
1060 reflections with *I* > 2σ(*I*)
*R*_int_ = 0.065

#### Refinement

*R*[*F* ^2^ > 2σ(*F* ^2^)] = 0.032
*wR*(*F* ^2^) = 0.081
*S* = 1.10
1355 reflections
53 parameters
H-atom parameters constrained
Δρ_max_ = 1.48 e Å^−3^
Δρ_min_ = −2.34 e Å^−3^

### 

Data collection: *IPDS Program Package* (Stoe & Cie, 1998 ▶); cell refinement: *IPDS Program Package*; data reduction: *IPDS Program Package*; program(s) used to solve structure: *SHELXS97* (Sheldrick, 1997 ▶); program(s) used to refine structure: *SHELXL97* (Sheldrick, 1997 ▶); molecular graphics: *DIAMOND* (Brandenburg, 1999 ▶); software used to prepare material for publication: *CIFTAB* in *SHELXTL* (Bruker, 1998 ▶).

## Supplementary Material

Crystal structure: contains datablocks I, global. DOI: 10.1107/S1600536807067682/si2066sup1.cif

Structure factors: contains datablocks I. DOI: 10.1107/S1600536807067682/si2066Isup2.hkl

Additional supplementary materials:  crystallographic information; 3D view; checkCIF report

## Figures and Tables

**Table:** 

W1—S1	2.1862 (13)
W1—S2	2.199 (2)
W1—S3	2.2010 (18)

**Table:** 

S1^i^—W1—S1	108.46 (7)
S1—W1—S2	108.62 (5)
S1—W1—S3	110.45 (5)
S2—W1—S3	110.18 (8)

Symmetry code: (i) 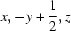.

**Table 2:** Hydrogen-bond geometry (Å, °)

*D*—H⋯*A*	*D*—H	H⋯*A*	*D*⋯*A*	*D*—H⋯*A*
N1—H1N1⋯S1^ii^	0.91	2.55	3.328 (6)	144
N1—H1N1⋯S3^ii^	0.91	2.90	3.5623 (12)	131
N1—H2N1⋯S2	0.91	2.40	3.227 (7)	152
N1—H2N1⋯S3	0.91	2.83	3.390 (7)	121
N2—H1N2⋯S1	0.91	2.77	3.468 (6)	135
N2—H1N2⋯S1^iii^	0.91	2.95	3.502 (6)	121
N2—H1N2⋯S2^iii^	0.91	2.96	3.5491 (12)	124
N2—H2N2⋯S3^iv^	0.91	2.64	3.464 (7)	151
C1—H1*A*⋯S1^v^	0.98	2.97	3.736 (8)	135
C1—H1*B*⋯S2^vi^	0.96	2.89	3.589 (10)	131

Symmetry codes: (ii) 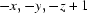; (iii) 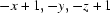; (iv) 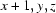; (v) 
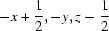
; (vi) 
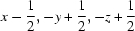
.

**Fig. 1.**
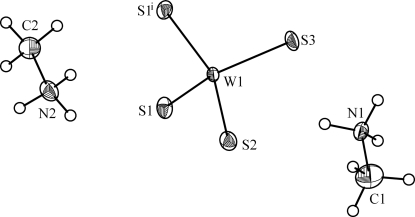
Perspective view of the title compound showing the atom-labelling scheme. Displacement ellipsoids are drawn at the 50% probability level. Symmetry code: (i) x, -y + 1/2, z

**Fig. 2.**
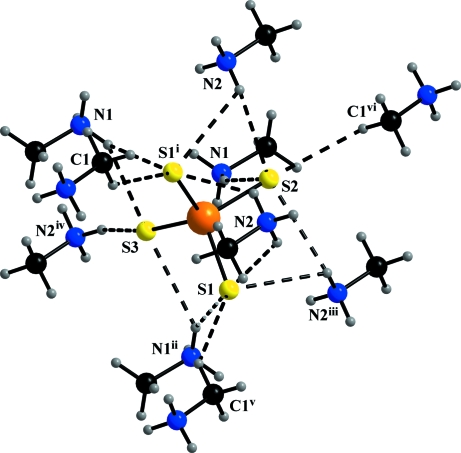
A view of the surroundings of the [WS4]2- anion showing its linking to ten different organic cations with the aid of N—H···S and C—H···S interactions. Symmetry codes: (i) x, -y + 1/2, Z (ii) -x, -y, -z + 1; (iii) -x + 1, -y, -z + 1 (iv) x + 1, y, z (v) -x + 1/2, -y, z - 1/2 (vi) x - 1/2, -y + 1/2, -z + 1/2

**Fig. 3.**
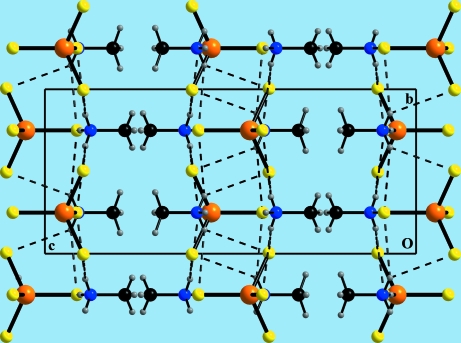
A view of the crystallographic packing of the title compound along the a axis, showing the organization of the organic cations and [WS4]2- anions. H-bonds are shown as broken lines.

## supplementary crystallographic information

### Comment

In continuation of our recent reports on the synthesis and structural characterization of organic ammonium tetrasulfidotungstates (Srinivasan, Naik *et al.*, 2007 and related literature cited therein; Srinivasan, Girkar & Raghavaiah 2007) we describe herein the structure of a new organic [WS_4_]^2-^ compound containing methylammonium as counter cation.

The title compound is isostructural with (NH_4_)_2_[WS_4_], (Srinivasan *et al.*, 2004), Cs_2_[WS_4_] (Srinivasan, Näther & Bensch 2007), Rb_2_[WS_4_] (Yao & Ibers 2004), and (CH_6_N)_2_[MoS_4_] (Srinivasan,, Näther *et al.*, 2006). The structure consists of a discrete tetrahedral [WS_4_]^2-^ ion and two crystallographically independent methylammonium cations (Fig. 1) all of which are located on crystallographic mirror planes so that each half of these ions make up the asymmetric unit. The bond lengths and bond angles of the organic cations are in good agreement with the reported values for the isotypic Mo compound. The WS_4_ tetrahedron is slightly distorted with S—W—S angles between 108.46 (7) and 110.45 (5) ° (Table 1). The W—S bond lengths range from 2.1862 (13) to 2.2010 (18) Å with an average value of 2.1931 Å. The observed difference Δ between the longest and the shortest W—S bonds of 0.0148 Å in the title compound is slightly shorter than the Δ value of 0.0199 Å in the analogous Mo compound (CNH_6_)_2_[MoS_4_] (Srinivasan, Näther *et al.*, 2006).

A scrutiny of the structure reveals that each [WS_4_]^2-^ is hydrogen bonded to ten symmetry related organic cations *via* several weak N—H···S and C—H···S interactions (Fig.2). These weak interactions can explain the observed distinct W—S bond distances. In general, unshared H-bonds have a considerably stronger effect than bifurcated ones, while the effect of trifurcated H-bonds is quite weak (Jeffrey, 1997). The atoms H1N1 and H2N1 are involved in bifurcated H-bonding while H1N2 makes a trifurcated H-bond, and H2N2 forms an unshared H-bond. Although short S···H contacts are observed at 2.40 and 2.55 Å for S2 and S1 respectively, these are relatively weaker in view of the bifurcated nature of H-bonding and hence do not cause much lengthening of these W—S bonds. The weakness of the trifurcated H-bond can also be evidenced from the observed longer S···H distances accompanied by smaller N—H···S angles. S3 is involved in a singly unshared H-bond at a distance of 2.64 Å. The effect of this contact is stronger than all the other contacts and can explain the elongation of the W—S3 bond, the longest observed W—S distance for the title compound. In all ten short S···H contacts ranging from 2.40 to 2.97 Å, all of which are shorter than the sum of their van der Waals radii (Bondi, 1964) are observed (Table 2). As a result of H-bonding, the organic cations are organized such that the ammonium groups always point towards the S atoms of tetrasulfidotungstate as can be seen in the sequence in the crystallographic *bc* plane *viz*. WS_4_··· H_3_NMe··· MeNH_3_··· WS_4_··· and so on (Fig. 3).

### Experimental

(NH_4_)_2_[WS_4_] (1 g) was dissolved in 40% methylamine (5 ml) and water (2 ml) and filtered. The clear yellow filtrate was left undisturbed for crystallization. After a day crystalline blocks of the title compound separated. The product was filtered, washed with ice-cold water (1 ml), followed by 2-propanol (5 ml) and diethyl ether (10 ml) and dried. Yield: 1.1 g.

### Refinement

All H atoms were located in difference map but were positioned with idealized geometry ((CH_3_ and NH_3_ allowed to rotate but not to tip) with 0.98 and 0.96 Å (methyl) and N—H = 0.91 Å) and were refined using a riding model, with *U*_iso_(H) fixed at 1.5*U*_eq_(CH_3_ and NH_3_). The largest peak in the residual electron density map of 1.48 e Å^-3^ is located at a distance of 0.09 Å from W1 and the deepest hole of -2.34 e Å^-3^ is located at a distance of 0.83 Å from W1.

### Crystal data

**Table:**

(CH_6_N)_2_[WS_4_]	*F*_000_ = 704
*M**_r_* = 376.23	*D*_x_ = 2.373 Mg m^−^^3^
Orthorhombic, *P**n**m**a*	Mo *K*α radiation λ = 0.71073 Å
Hall symbol: -P 2ac 2n	Cell parameters from 8000 reflections
*a* = 9.5591 (10) Å	θ = 1.9–28.2º
*b* = 7.0006 (7) Å	µ = 11.70 mm^−^^1^
*c* = 15.734 (2) Å	*T* = 170 (2) K
*V* = 1052.9 (2) Å^3^	Block, yellow
*Z* = 4	0.12 × 0.06 × 0.04 mm

### Data collection

**Table:**

Stoe IPDS1 diffractometer	1355 independent reflections
Radiation source: fine-focus sealed tube	1060 reflections with *I* > 2σ(*I*)
Monochromator: graphite	*R*_int_ = 0.065
*T* = 170(2) K	θ_max_ = 27.9º
φ scans	θ_min_ = 2.5º
Absorption correction: Numerical(X-SHAPE; Stoe & Cie, 1998)	*h* = −12→12
*T*_min_ = 0.441, *T*_max_ = 0.631	*k* = −9→9
9418 measured reflections	*l* = −20→20

### Refinement

**Table:**

Refinement on *F*^2^	Hydrogen site location: inferred from neighbouring sites
Least-squares matrix: full	H-atom parameters constrained
*R*[*F*^2^ > 2σ(*F*^2^)] = 0.032	*w* = 1/[σ^2^(*F*_o_^2^) + (0.045*P*)^2^ + 1.2501*P*] where *P* = (*F*_o_^2^ + 2*F*_c_^2^)/3
*wR*(*F*^2^) = 0.081	(Δ/σ)_max_ < 0.001
*S* = 1.10	Δρ_max_ = 1.48 e Å^−^^3^
1355 reflections	Δρ_min_ = −2.34 e Å^−^^3^
53 parameters	Extinction correction: SHELXL97 (Sheldrick, 1997), Fc^*^=kFc[1+0.001xFc^2^λ^3^/sin(2θ)]^-1/4^
Primary atom site location: structure-invariant direct methods	Extinction coefficient: 0.0022 (3)
Secondary atom site location: difference Fourier map	

### Special details

**Table:**

Geometry. All e.s.d.'s (except the e.s.d. in the dihedral angle between two l.s. planes) are estimated using the full covariance matrix. The cell e.s.d.'s are taken into account individually in the estimation of e.s.d.'s in distances, angles and torsion angles; correlations between e.s.d.'s in cell parameters are only used when they are defined by crystal symmetry. An approximate (isotropic) treatment of cell e.s.d.'s is used for estimating e.s.d.'s involving l.s. planes.
Refinement. Refinement of *F*^2^ against ALL reflections. The weighted *R*-factor *wR* and goodness of fit *S* are based on *F*^2^, conventional *R*-factors *R* are based on *F*, with *F* set to zero for negative *F*^2^. The threshold expression of *F*^2^ > σ(*F*^2^) is used only for calculating *R*-factors(gt) *etc*. and is not relevant to the choice of reflections for refinement. *R*-factors based on *F*^2^ are statistically about twice as large as those based on *F*, and *R*- factors based on ALL data will be even larger.

### Fractional atomic coordinates and isotropic or equivalent isotropic displacement parameters (Å^2^)

**Table:**

	*x*	*y*	*z*	*U*_iso_*/*U*_eq_	
W1	0.24968 (3)	0.2500	0.552442 (15)	0.01100 (14)	
S3	0.02612 (18)	0.2500	0.58592 (13)	0.0191 (4)	
S2	0.2751 (2)	0.2500	0.41353 (13)	0.0209 (4)	
S1	0.35291 (14)	−0.00338 (18)	0.60404 (9)	0.0204 (3)	
N1	−0.0570 (7)	0.2500	0.3764 (4)	0.0178 (13)	
H1N1	−0.1078	0.1449	0.3909	0.027*	
H2N1	0.0246	0.2500	0.4063	0.027*	
C1	−0.0349 (12)	0.2500	0.2832 (6)	0.041 (2)	
H1A	0.0168	0.1361	0.2652	0.061*	
H1B	−0.1279	0.2500	0.2601	0.061*	
N2	0.6638 (7)	0.2500	0.5893 (4)	0.0233 (14)	
H1N2	0.6161	0.1449	0.5715	0.035*	
H2N2	0.7517	0.2500	0.5672	0.035*	
C2	0.6709 (10)	0.2500	0.6828 (5)	0.0282 (18)	
H2A	0.7249	0.3626	0.7000	0.042*	
H2B	0.5836	0.2500	0.7155	0.042*	

### Atomic displacement parameters (Å^2^)

**Table:**

	*U*^11^	*U*^22^	*U*^33^	*U*^12^	*U*^13^	*U*^23^
W1	0.00838 (19)	0.01006 (18)	0.01455 (19)	0.000	−0.00081 (11)	0.000
S3	0.0086 (8)	0.0257 (9)	0.0230 (9)	0.000	0.0029 (8)	0.000
S2	0.0157 (9)	0.0286 (9)	0.0183 (9)	0.000	0.0028 (8)	0.000
S1	0.0166 (6)	0.0131 (5)	0.0316 (6)	0.0016 (5)	−0.0057 (6)	0.0043 (5)
N1	0.016 (3)	0.016 (3)	0.021 (3)	0.000	−0.006 (3)	0.000
C1	0.048 (6)	0.058 (6)	0.016 (4)	0.000	0.003 (4)	0.000
N2	0.017 (3)	0.025 (3)	0.028 (4)	0.000	0.005 (3)	0.000
C2	0.027 (5)	0.037 (4)	0.021 (4)	0.000	0.002 (4)	0.000

### Geometric parameters (Å, °)

**Table:**

W1—S1^i^	2.1862 (13)	C1—H1A	0.98
W1—S1	2.1862 (13)	C1—H1B	0.96
W1—S2	2.199 (2)	N2—C2	1.473 (11)
W1—S3	2.2010 (18)	N2—H1N2	0.91
N1—C1	1.482 (10)	N2—H2N2	0.91
N1—H1N1	0.91	C2—H2A	0.98
N1—H2N1	0.91	C2—H2B	0.98
			
S1^i^—W1—S1	108.46 (7)	N1—C1—H1A	111.0
S1^i^—W1—S2	108.62 (5)	N1—C1—H1B	104.1
S1—W1—S2	108.62 (5)	H1A—C1—H1B	110.9
S1^i^—W1—S3	110.45 (5)	C2—N2—H1N2	109.4
S1—W1—S3	110.45 (5)	C2—N2—H2N2	109.8
S2—W1—S3	110.18 (8)	H1N2—N2—H2N2	110.2
C1—N1—H1N1	108.8	N2—C2—H2A	107.4
C1—N1—H2N1	112.8	N2—C2—H2B	119.0
H1N1—N1—H2N1	109.2	H2A—C2—H2B	107.7

Symmetry codes: (i) *x*, −*y*+1/2, *z*.

### Hydrogen-bond geometry (Å, °)

**Table:**

*D*—H···*A*	*D*—H	H···*A*	*D*···*A*	*D*—H···*A*
N1—H1N1···S1^ii^	0.91	2.55	3.328 (6)	144
N1—H1N1···S3^ii^	0.91	2.90	3.5623 (12)	131
N1—H2N1···S2	0.91	2.40	3.227 (7)	152
N1—H2N1···S3	0.91	2.83	3.390 (7)	121
N2—H1N2···S1	0.91	2.77	3.468 (6)	135
N2—H1N2···S1^iii^	0.91	2.95	3.502 (6)	121
N2—H1N2···S2^iii^	0.91	2.96	3.5491 (12)	124
N2—H2N2···S3^iv^	0.91	2.64	3.464 (7)	151
C1—H1A···S1^v^	0.98	2.97	3.736 (8)	135
C1—H1B···S2^vi^	0.96	2.89	3.589 (10)	131

Symmetry codes: (ii) −*x*, −*y*, −*z*+1; (iii) −*x*+1, −*y*, −*z*+1; (iv) *x*+1, *y*, *z*; (v) −*x*+1/2, −*y*, *z*−1/2; (vi) *x*−1/2, −*y*+1/2, −*z*+1/2.
